# Multidrug-resistant gram-negative bacteria colonization of healthy US military personnel in the US and Afghanistan

**DOI:** 10.1186/1471-2334-13-68

**Published:** 2013-02-05

**Authors:** Todd J Vento, David W Cole, Katrin Mende, Tatjana P Calvano, Elizabeth A Rini, Charla C Tully, Wendy C Zera, Charles H Guymon, Xin Yu, Kristelle A Cheatle, Kevin S Akers, Miriam L Beckius, Michael L Landrum, Clinton K Murray

**Affiliations:** 1 Brooke Army Medical Center/San Antonio Military Medical Center, Fort Sam Houston, TX, USA; 2 Uniformed Services University of the Health Sciences, Bethesda, MD, USA; 3 Blanchfield Army Community Hospital, Fort Campbell, KY, USA; 4 Infectious Disease Clinical Research Program, Uniformed Services University of the Health Sciences, Bethesda, MD, USA; 5 United States Army Institute of Surgical Research, Fort Sam Houston, TX, USA

**Keywords:** Deployment, Malaria chemoprophylaxis, Environment exposure, ESBL-production, *Escherichia coli*

## Abstract

**Background:**

The US military has seen steady increases in multidrug-resistant (MDR) gram-negative bacteria (GNB) infections in casualties from Iraq and Afghanistan. This study evaluates the prevalence of MDR GNB colonization in US military personnel.

**Methods:**

GNB colonization surveillance of healthy, asymptomatic military personnel (101 in the US and 100 in Afghanistan) was performed by swabbing 7 anatomical sites. US-based personnel had received no antibiotics within 30 days of specimen collection, and Afghanistan-based personnel were receiving doxycycline for malaria chemoprophylaxis at time of specimen collection. Isolates underwent genotypic and phenotypic characterization.

**Results:**

The only colonizing MDR GNB recovered in both populations was *Escherichia coli* (p=0.01), which was seen in 2% of US-based personnel (all perirectal) and 11% of Afghanistan-based personnel (10 perirectal, 1 foot+groin). Individuals with higher off-base exposures in Afghanistan did not show a difference in overall GNB colonization or MDR *E. coli* colonization, compared with those with limited off-base exposures.

**Conclusion:**

Healthy US- and Afghanistan-based military personnel have community onset-MDR *E. coli* colonization, with Afghanistan-based personnel showing a 5.5-fold higher prevalence. The association of doxycycline prophylaxis or other exposures with antimicrobial resistance and increased rates of MDR *E. coli* colonization needs further evaluation.

## Background

Antimicrobial resistance of commonly recovered bacteria is a global threat [1]. The global impact of these pathogens is demonstrated by incident infections and colonization with multidrug-resistant (MDR) bacteria in individuals returning from leisure travel [2], the identification of novel resistance genes in patients seeking medical care in other countries [3], the finding of novel resistance genes in environmental sources such as drinking water [4], and increasing reports of community-acquired MDR-GNB infections, such as ESBL-producing *Escherichia coli* urinary tract infections [5].

Since the beginning of combat operations in Iraq and Afghanistan, the US military healthcare system has dealt with steady increases in resistant organisms isolated from wounded service members, similar to experiences during international disaster relief and humanitarian missions [6-10]. Potential sources of these MDR organisms include inoculation of the wound with host normal flora at the time of injury, wound contamination from environmental soil or water organisms, or nosocomial transmission during evacuation through medical facilities. Previous studies have supported colonized local national patients in Afghanistan as a likely source of cross-contamination in deployed US hospitals, but to date have not targeted other potential mechanisms of acquisition, such as MDR bacteria acquisition from environmental soil exposure or preexisting colonization of US casualties who are injured [9,11]. Personnel deployed to Afghanistan are also under antimicrobial pressure with daily antimalarial chemoprophylaxis, which might alter an individual’s colonizing pathogens. Given the reports of increasing MDR pathogens isolated from healthy people and from environmental sources such as drinking water and food products, we evaluated colonization rates of two distinct populations of military personnel: healthy US military trainees who had not been deployed and healthy, uninjured US service members currently deployed to Afghanistan. The purpose of the study was to determine the prevalence of MDR-GNB across multiple anatomic sites in geographically distinct environments with different environmental exposures and antimicrobial pressures.

## Methods

### Participants

The two populations under study included 101 non-deployed healthy active duty service members in San Antonio, Texas and 100 healthy active duty service members deployed to a single province in Afghanistan. Participants were recruited after they presented to their respective outpatient medical clinic for acute, non-urgent/emergent care with no active infection. All participants were 18 years or older and provided written informed consent for study participation. The non-deployed participants were excluded if they had recent overseas travel (within 6 months), overseas deployment (within 6 months), or antibiotic use (within 30 days). The deployed personnel had been in Afghanistan for approximately 7 months and had been prescribed doxycycline for malaria chemoprophylaxis (100 mg orally daily). The protocols were approved by the Brooke Army Medical Center and the United States Army Medical Research and Material Command Institutional Review Boards.

### Surveillance cultures

#### Troop medical clinic, San Antonio, TX

The research team performed culture collection from the nares, oropharynx, axilla, groin, web spaces of dominant hand, web spaces of the foot, and perirectal area using a pre-moistened swab (Copan, Stuart liquid media culture, Copan Inc., Brescia, Italy). Swabs were transported to the laboratory immediately and plated onto Trypticase™ Soy Agar with 5% sheep blood and MacConkey agar in order to isolate all GNB colonies. After incubation at 35°C, colonies with morphology consistent GNB growing on sheep blood agar and MacConkey agar plates were subcultured onto sheep blood agar in order to ensure culture purity. Isolates were frozen at -80°C in Trypticase™ Soy Broth (TSB) with 15% glycerol. Specimen collection period was from May to June 2011.

#### Acute care clinic in Afghanistan

Samples collected from deployed participants in Afghanistan were collected using BD CultureSwab™ MaxV(+) (Becton Dickinson, Franklin Lakes, NJ), which contain Amies medium and a unique blend of non-animal proteins embedded into the swab fibers, providing additional nutrients for the survival of microorganisms during transport. The same sample collection sites and techniques as described for the US study site were used. Specimens were collected during August 2011 and shipped within 14 days of collection. Identification and storage was performed as described above.

#### Antimicrobial susceptibility testing

Isolates underwent automated testing for identification and susceptibility using the BD Phoenix Automated Microbiology System (Becton Dickinson and Company, Franklin Lakes, NJ) using PMIC/ID-107, NMIC/ID-123 and NMIC/ID-139 panels. Epsilometer-test (Etest^®^) (bioMerieux, Durham, NC, USA) was used for antimicrobial testing of doxycycline, tetracycline and minocycline. Minocycline breakpoints were defined in the package insert as susceptible ≤ 4 μg/ml, intermediate 8 μg/ml, and resistant ≥ 16 μg/ml; values falling between two-fold dilutions were rounded up to the next two-fold value before categorization. Moxifloxacin antimicrobial activity against GNB was defined as susceptible ≤1 μg/ml, intermediate 2–4 μg/ml, and resistant >4 μg/ml because no Clinical and Laboratory Standard Institute (CLSI) or Phoenix minimum inhibitory concentration (MIC) interpretive criteria were available. Confirmation testing of ESBL status was performed using the disk diffusion method per CLSI recommendations [12]. A multidrug resistant (MDR) organism was defined as any ESBL-producing bacteria, or if resistant to all tested antimicrobials in 3 or more antimicrobial classes (penicillins/cephalosporins, carbapenems, aminoglycosides, and quinolones) not including tetracyclines [13].

#### Molecular testing

Isolates underwent pulsed-field gel electrophoresis (PFGE) using the Food and Drug Administration (FDA) protocol for GNB. Clonality was assessed using the commercial software BioNumerics (Applied Maths Inc., Austin, TX) and defined by 85% similarity for the GNB [14]. Those isolates identified by the microbiology laboratory as likely to produce ESBL enzymes were further typed to identify the following specific classes of ESBL enzymes: CTX-M, SHV, and TEM. Representative isolates with CTX-M group and TEM underwent sequencing to confirm the gene and type of CTX-M or TEM present [15]. Isolates were screened for tetracycline resistance genes *tet*(A), *tet*(B), *tet*(C), *tet*(D), *tet*(E), and *tet*(M).

### Statistical analysis

Categorical values were compared using Pearson χ-square or Fisher’s Exact Test analysis and continuous variables using Mann–Whitney U test. All statistical operations were performed using SPSS (IBM^®^ SPSS^®^ Statistics Version 19). All statistical tests were two-tailed and a p-value of <0.05 was considered statistically significant.

## Results

### Demographics of study participants

Over 1400 total swab samples were obtained from the two different study populations. There were 704 swabs obtained from the 101 US-based study participants; two participants refused collection from the perirectal site and one refused collection from the oropharyngeal site. The mean age was 23 years, 69% were male, 85% born in the United States, and 81% were Army Soldiers. The mean time of active duty military service was nine (SD 10.3) months, and mean time at San Antonio, TX was three (SD 2.6) months. Collection of samples from 100 healthy participants deployed to Afghanistan yielded 700 samples. Median age was 23 years; all participants were of male gender. All personnel were in the Army and 84% of them performed foot patrols outside of the base possibly predisposing them to more local environmental and population exposures.

### Gram negative bacteria from US-based subjects

**Figure 1:**
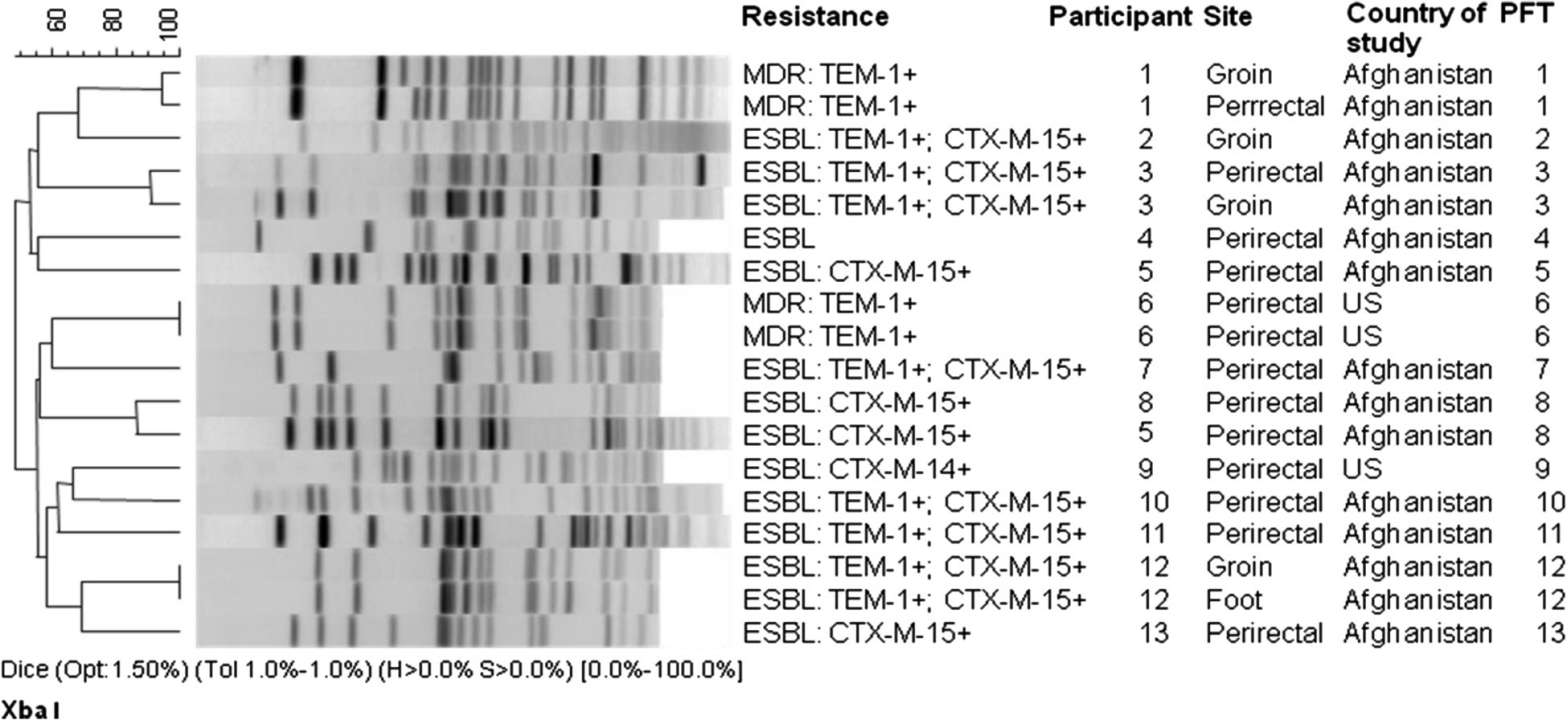
**Pulsed-field gel electrophoresis pattern, country of study, and body region of ESBL-producing and multidrug-resistant (MDR) *Escherichia coli *along with TEM and CTX-M mechanisms of resistance**

The samples yielded 191 GNB from 85 participants, of which only three isolates (all perirectal) from two participants were found to be MDR-GNB (Tables 1, 2). The two perirectal MDR *E. coli* isolates from the single patient were phenotypically different in appearance during initial culture, necessitating further testing. Five participants were colonized with *Stenotrophomonas maltophilia* and one with *Burkholderia cepacia*, which are considered MDR pathogens by some criteria but not ours. The resistant *E. coli* included one ESBL-producing isolate [one participant with one pulsed-field type (PFT)] and two non-ESBL-producing isolates (one participant with one PFT) (Figure 1, Table 3 and Table 4). The two non-ESBL-producing isolates’ antibiograms were identical, with slightly more antimicrobial resistance in comparison to the other study participant’s isolate (Tables 3, 4). The CTX-M-14 resistance gene (confirmed by sequencing) was recovered in the single ESBL-producing isolate, and TEM was found in the two non-ESBL-producing MDR *E. coli* isolates (sequences matched TEM-1) (Figure 1).

**Table 1:** Number of participants colonized with gram-negative bacteria by body site of 101^* ^**non-deployed, healthy US military service members presenting to a troop medical clinic**

Isolate**	**Nares**	**Oropharynx**	**Axilla**	**Groin**	**Hand**	**Foot**	**Perirectal**	**Number of participants colonized**
Total MDR bacteria (all *Escherichia coli*)	0	0	0	0	0	0	2 (3)	2 (3)
Total non-MDR bacteria	4 (4)	14 (17)	11 (12)	9 (14)	7 (7)	27 (35)	65 (99)	85 (188)
*Acinetobacter baumannii-calcoaceticus *complex	0	1 (1)	0	0	1 (1)	4 (4)	2 (2)	8 (8)
*Acinetobacter lwoffii*	0	1 (1)	0	1 (1)	0	4 (5)	2 (2)	8 (9)
*Alcaligenes *species	0	1 (1)	0	0	1 (1)	2 (2)	1 (1)	5 (5)
*Citrobacter *species	0	0	0	0	0	0	11 (11)	11 (11)
*Enterobacter aerogenes*	1 (1)	0	2 (2)	1 (1)	0	0	1 (1)	5 (5)
*Enterobacter cloacae*	0	1 (1)	0	0	1 (1)	0	4 (4)	6 (6)
*Escherichia coli*	2 (2)	0	2 (3)	1 (1)	0	0	49 (54)	49 (60)
*Klebsiella oxytoca*	0	1 (1)	0	1 (1)	0	0	2 (3)	2 (5)
*Klebsiella pneumoniae*	0	1 (1)	1 (1)	2 (2)	0	1 (1)	8 (8)	12 (13)
*Proteus* species	0	0	1 (1)	0	0	1 (1)	0	2 (2)
*Pseudomonas aeruginosa*	0	4 (4)	0	0	0	2 (2)	0	5 (6)
*Serratia marcescens*	1 (1)	1 (1)	4 (4)	1 (1)	2 (2)	2 (2)	1 (1)	9 (12)
*Stenotrophomonas maltophilia*	0	2 (2)	0	0	0	3 (3)	1 (1)	5 (6)
Other***	0	4 (4)	1 (1)	4 (7)	2 (2)	12 (15)	11 (11)	34 (40)

Number of isolates in parentheses.

*100 participants were swabbed at the oropharynx and 99 individuals were swabbed in the perirectal sites.

**Participants can be colonized with multiple bacteria or clones of bacteria.

***Other isolates include: *Acinetobacter *species (2)*, Acinetobacter haemolyticus *(2)*, Burkholderia cepacia, Burkholderia gladioli, Comamonas testosteroni, Enterobacter hormaechei, Leclericia adecarboxylata, Moraxella *species (6), *Morganella morganii *(3), *Ochrobactrum anthropi *(2), *Pantoea agglomerans *(6)*, Providencia alcalifaciens, Pseudomonas *species*, Pseudomonas fluorescens, Pseudomonas luteola, Pseudomonas oryzihabitans* (3)*, Pseudomonas putida, Pseudomonas stutzeri* (2)*, Rhizobium radiobacter, Shewanella putrefaciens, Shigella flexneri (2).*

**Table 2:** Comparison of participants* from the US and Afghanistan study sites with gram-negative bacteria

	**US (n=101)**	**Afghanistan (n=100)**	**P value**
Age (median, IQR)	22 (20,25)	23 (22,25.8)	0.07
Gender- males	69	100	<0.01
Total colonized	85	84	0.98
Nares	4	12	0.04
Oropharynx	14	8	0.26
Axilla	11	13	0.65
Groin	9	23	<0.01
Hand	7	12	0.22
Foot	27	13	0.15
Perirectal	66	70	0.48
Total multidrug resistant (MDR) colonized	2	11	0.01
Groin	0	4	0.12
Foot	0	1	0.50
Perirectal	2	9	0.03
All bacteria			
*Acinetobacter baumannii-calcoaceticus *complex	8	4	0.24
*Acinetobacter lwoffii*	8	13	0.24
*Citrobacter *species	11	5	0.19
*Enterobacter aerogenes*	5	9	0.28
*Enterobacter cloacae*	6	3	0.50
*Escherichia coli*	51	67	0.02
*Klebsiella pneumoniae*	12	20	0.12
*Pseudomonas aeruginosa*	5	2	0.45
*Serratia marcescens*	9	1	0.02
*Stenotrophomonas maltophilia*	5	0	0.06
Total sites colonized			0.44
0	16	16	
1	48	36	
2	24	31	
3	10	15	
4	3	2	
Total gram-negative rod bacteria isolates			0.62
0	16	16	
1	32	24	
2	23	22	
3	17	21	
4	7	10	
5	4	5	
6	0	1	
7	2	0	
10	0	1	

*Participants can be colonized with multiple bacteria or clones of bacteria.

**Table 3:** **Antimicrobial resistance of multidrug-resistant *Escherichia coli***

**Participant**	**Country of study**	**Resistance profile**		**Antimicrobial Resistance Testing**																	
			**Amikacin**	**Ampicillin- Sulbactam**	**Aztreonam**	**Cefepime**	**Cefoxitin**	**Ceftazidime**	**Ceftriaxone**	**Cefuroxime**	**Ciprofloxacin**	**Ertapenem**	**Gentamicin**	**Imipenem**	**Levofloxacin**	**Meropenem**	**Moxifloxacin**	**Nitrofurantoin**	**Piperacillin- Tazobactam**	**Tobramycin**	**Trimethoprim- Sulfamethoxazole**
6	US	MDR	S	R	R	S	R	R	R	R	R	S	R	S	R	S	R	S	R	I	R
6	US	MDR	S	R	R	S	R	R	R	R	R	S	R	S	R	S	R	S	R	I	R
9	US	ESBL	S	R	R	R	I	R	R	R	R	S	S	S	R	S	R	S	S	S	R
% susceptible by unique isolate per unique participant			100	0	0	50	0	0	0	0	0	100	50	100	0	100	0	100	50	50	0
1	Afghan	MDR	S	R	R	S	R	R	R	R	S	S	S	S	S	S	S	S	I	S	R
1	Afghan	MDR	S	R	R	S	R	R	R	R	S	S	S	S	S	S	S	S	I	S	R
2	Afghan	ESBL	S	R	R	R	I	R	R	R	R	S	R	S	R	S	R	S	S	R	R
3	Afghan	ESBL	S	R	R	R	S	R	R	R	S	S	S	S	S	S	S	S	S	S	R
3	Afghan	ESBL	S	R	R	R	S	R	R	R	S	S	S	S	S	S	S	S	I	S	R
4	Afghan	ESBL	S	R	R	R	I	R	R	R	S	S	S	S	S	S	S	S	S	S	S
5 (PFT 5)	Afghan	ESBL	S	R	R	R	S	R	R	R	S	S	S	S	S	S	S	S	S	S	R
5 (PFT 8)	Afghan	ESBL	S	R	R	R	S	R	R	R	R	S	S	S	R	S	R	S	S	R	R
7	Afghan	ESBL	S	R	R	R	S	R	R	R	R	S	S	S	R	S	R	I	S	S	R
8	Afghan	ESBL	S	R	R	R	S	R	R	R	R	S	S	S	R	S	R	S	S	R	R
10	Afghan	ESBL	S	R	R	R	S	R	R	R	S	S	S	S	S	S	S	I	S	S	R
11	Afghan	ESBL	S	R	R	R	S	R	R	R	R	S	S	S	R	S	R	S	S	S	R
12	Afghan	ESBL	S	R	R	R	S	R	R	R	S	S	S	S	S	S	I	S	S	S	R
12	Afghan	ESBL	S	R	R	R	S	R	R	R	S	S	S	S	S	S	I	S	S	S	R
13	Afghan	ESBL	S	R	R	R	S	R	R	R	R	S	S	S	R	S	R	S	S	R	R
% susceptible by unique isolate per unique participant			100	0	0	8	75	0	0	0	42	100	92	100	50	100	42	83	92	75	8

MDR- multidrug-resistant; ESBL- Extended spectrum beta-lactamase producing; PFT- pulsed-field type; S-Susceptible; R-Resistant; I-Intermediate.

**Table 4:** **Doxycycline, minocycline and tetracycline resistance and tetracycline resistance genes for **multidrug-resistant ***E. coli *isolates**

**Participant**	***tet*(A)**	***tet*(B)**	**Doxycycline**	**Minocycline**	**Tetracycline**
1	+	-	R	S	R
1	+	-	R	S	R
2	-	+	R	R	R
3	+	-	I	S	R
3	+	-	I	S	R
4	-	-	S	S	S
5 (PFT 5)	-	+	R	I	R
5 (PFT 8)	+	-	R	I	R
6	+	-	R	I	R
6	+	-	R	I	R
7	-	+	R	R	R
8	+	-	R	I	R
9	-	+	R	R	R
10	-	+	R	R	R
11	-	+	R	R	R
12	+	-	R	I	R
12	+	-	R	I	R
13	+	-	R	I	R

- no gene detected; + gene detected; R- resistant, I- intermediate, S-susceptible; PFT- pulsed-field type.

### Gram negative bacteria from Afghanistan-based subjects

The samples revealed 212 non-MDR GNB isolates from 84 participants, with 15 isolates from 11 participants being MDR pathogens (all *E. coli*). *Escherichia coli* colonized the greatest number of participants in this study population (Table 5). The 15 resistant *E. coli* isolates included 13 ESBL-producing bacteria. There were 11 different PFTs from 11 participants with MDR *E. coli* (Figure 1). One participant had two perirectal isolates, appearing phenotypically different on culture, with different PFTs. One of those perirectal isolates matched another participant’s perirectal isolate by PFGE. The two participants with matching PFT perirectal isolates had isolates with identical antimicrobial resistance and genetic resistance results. The two perirectal isolates from the same participant with different PFTs had different antimicrobial resistance and genetic resistance results. Different antimicrobial resistance patterns were described among the 11 participants’ isolates without a common resistance profile, except that all isolates were susceptible to amikacin and carbapenems; 80% of isolates (12 of 15) were susceptible to piperacillin-tazobactam, but all were resistant to ampicillin-sulbactam (Table 3). TEM was present in ten isolates (all isolates were sequenced revealing them to be TEM-1) and CTX-M-15 was detected in 12 isolates (two isolates were confirmed by sequencing) with eight isolates having both and one isolate with no identifiable resistance mechanisms (Figure 1). An analysis of isolates from those with prior deployments did not reveal a difference in colonization with any bacteria (17 of 21 with prior deployment versus 67 of 79 without prior deployment, p=0.74) or MDR *E. coli* (1 of 21 with prior deployment versus 10 of 79 without prior deployment, p=0.45). In addition, analysis of those with exposure outside of the base did not reveal a difference in colonization with any bacteria (71 of 84 outside of the base versus 13 of 16 not outside of the base, p=0.74) or MDR *E. coli* (10 of 84 outside of the base versus 1 of 16 not outside of the base, p=1.00).

**Table 5:** Number of participants with gram-negative bacteria by body site of 100 healthy US military service members deployed to Afghanistan who presented to an acute care clinic

Isolate*	**Nares**	**Oropharynx**	**Axilla**	**Groin**	**Hand**	**Foot**	**Perirectal**	**Number of participants colonized**
Total MDR bacteria (all *Escherichia coli*)	0	0	0	4 (4)	0	1 (1)	9 (10)	11 (15)
Total non-MDR bacteria	12 (13)	8 (10)	13 (14)	20 (27)	12 (12)	12 (14)	65 (107)	80 (197)
*Acinetobacter baumannii-calcoaceticus* complex	0	0	0	0	1 (1)	3 (3)	0	4 (4)
*Acinetobacter lwoffii*	0	0	3 (3)	2 (2)	5 (5)	5 (5)	1 (1)	13 (16)
*Alcaligenes* species	0	1 (1)	0	2 (2)	1 (1)	2 (2)	1 (1)	5 (7)
*Citrobacter* species	1 (1)	0	0	0	0	1 (1)	3 (3)	5 (5)
*Enterobacter aerogenes*	5 (6)	2 (2)	4 (4)	1 (1)	1 (1)	0	2 (2)	9 (16)
*Enterobacter cloacae*	0	1 (2)	0	1 (1)	0	1 (1)	0	3 (4)
*Escherichia coli*	2 (2)	0	3 (3)	12 (17)	0	0	57 (75)	59 (97)
*Klebsiella oxytoca*	3 (3)	0	0	0	0	0	0	3 (3)
*Klebsiella pneumoniae*	0	0	2 (2)	2 (2)	0	0	19 (20)	20 (24)
*Proteus* species	0	0	1 (1)	0	0	0	2 (2)	3 (3)
*Pseudomonas aeruginosa*	0	1 (1)	0	1 (1)	0	0	2 (2)	2 (4)
*Serratia marcescens*	1 (1)	1 (2)	0	0	0	0	0	1 (3)
*Stenotrophomonas maltophilia*	0	0	0	0	0	0	0	0
Other**	0	2 (2)	1 (1)	1 (1)	4 (4)	2 (2)	1 (1)	11 (11)

Number of isolates in ().

* Participants can be colonized with multiple bacteria or clones of bacteria.

**Other isolates includes: *Comamonas testosteroni, Enterobacter gergoviae, Moraxella *species (2), *Pantoea agglomerans *(3)*, Pseudomonas *species*, Pseudomonas putida, Serratia liquefaciens, Sphingomonas paucimobilis*.

#### Comparison of US (non-deployed) and Afghanistan (deployed) troop colonization

For the entire population, the presence of MDR *E. coli* colonization was associated with geographic study site (2% US site versus 11% Afghanistan site, p=0.01) as was the presence of non-MDR *E. coli* colonization (49% US versus 59% Afghanistan, p=0.04). However, the number of participants colonized, number of colonized anatomic sites, number of co-colonizing pathogens, and type of co-colonizing pathogen were not associated with MDR *E. coli* colonization. The two study populations had similar prevalence values by different anatomic site, except for the groin area: 23% of Afghanistan subjects versus 9% of US subjects were colonized with GNB in the groin, p<0.01 (Table 2). The participants were also colonized with similar pathogens overall, with the following exceptions: Afghanistan-based participants had more *E. coli* isolates (67% versus 51%, p=0.02), while US based study participants had more *Serratia* (9% versus 1% p=0.02).

Among MDR *E. coli* isolates, all participants from the US had isolates resistant to tetracycline and doxycycline; one subject with one isolate resistant to minocycline and the other subjects with two isolates with intermediate susceptibility to minocycline. Ten of 11 Afghanistan-based participants had isolates resistant to tetracycline with incongruent doxycycline and minocycline susceptibilities, but high overall doxycycline resistance (Table 4). The two US clonal isolates from the same participant were resistant to tetracycline, with one isolate showing the *tet*(A) resistance gene and the other isolate having the *tet*(B) gene (Table 4)*.* Despite the presence of *tet*(A) or *tet*(B) for all MDR *E. coli*, four isolates retained susceptibility to minocycline from the Afghanistan population (Table 4). All isolates were resistant to ampicillin-sulbactam while the vast majority was susceptible to piperacillin-tazobactam. There were no overlapping PFGE patterns among isolates from the US and Afghanistan populations (Figure 1).

There were 49 US participants colonized with 60 isolates of non-MDR *E. coli* (two nare, three axilla, one groin, and 54 perirectal, for a total of 48 distinct PFTs) and 58 Afghanistan participants colonized with 97 non-MDR *E. coli* (two nare, three axilla, 17 groin, and 75 perirectal, for a total of 64 distinct PFTs). Comparing US isolates to Afghanistan isolates, there were significant differences in antimicrobial susceptibilities to ampicillin (72% vs. 49%, p<0.01), ampicillin-sulbactam (73% vs. 54%, p<0.01), ciprofloxacin (97% vs. 88%, p=0.05), and trimethoprim-sulfamethoxazole (82% vs. 44%, p<0.01). E-test^®^ results also showed differences in susceptibility to both tetracycline/doxycycline (80% vs. 33%, p<0.01) and minocycline (85% vs. 46%, p<0.01).

The TEM beta-lactamase enzyme was detected but not phenotypically expressed in 17 non-MDR *E. coli* isolates from 14 US participants and 45 isolates from 30 Afghanistan participants (p=0·02) (3 isolates sequenced as TEM-1 and 10 randomly selected isolates confirmed non-ESBL-producers by disk diffusion testing). The presence of TEM was associated with resistance to ampicillin (92% TEM present versus 4% TEM absent, p<0.01), ampicillin-sulbactam (100% TEM present versus 4% TEM absent, p<0.01), cefazolin (35% TEM present versus 3% TEM absent, p<0.01), trimethoprim-sulfamethoxazole (68% TEM present versus 23% TEM absent, p<0.01), both tetracycline and doxycycline (81% TEM present versus 27% TEM absent, p<0.01), and minocycline (55% TEM present versus 27% TEM absent, p<0.01). The *tet*(A) gene was identified in five US isolates and 50 Afghanistan isolates (p<0.01) and *tet*(B) in seven US isolates and15 Afghanistan isolates (p=0.5). One US-based participant grew two isolates, with *tet*(A) and *tet*(B) resistance genes identified, but no other *tet* resistance genes were recovered. Overall, there was no clonality associated with antimicrobial resistance.

## Discussion

Multidrug-resistant bacteria are present throughout the world [1,3,4]. The global movement of these pathogens through leisure travel, medical tourism, and military casualty movement is concerning [2-4]. For military personnel, one proposed source of MDR bacteria has been pathogen introduction into deployed military treatment facilities through host nation patients, with subsequent nosocomial transmission to US personnel. However, most of the data supporting this conclusion were from studies completed over five years ago and focused on MDR *Acinetobacter*[8,9]. During 2009 and 2010, increasing numbers of casualties were noted to be colonized with ESBL-producing *E. coli*, although no source has been elucidated for this possible epidemiological shift in MDR organisms [16]. In this study, 2% of US-based personnel (without recent antibiotic exposure, overseas travel, or active infection) were colonized with MDR *E. coli* in the perirectal region, whereas Afghanistan-based personnel showed an 11% colonization prevalence of MDR *E. coli*. This finding demonstrates significantly higher (5.5-fold, p<0.01) colonization in individuals based primarily on their deployed status, as their prior healthcare system exposure was also minimal. This finding is consistent with a prior study demonstrating prevalent and incident GNR colonization in recently deployed, hospitalized individuals compared to non-deployed hospitalized patients [17]. In contrast to this previous study, which demonstrated a 60% clonality of incident MDR *A. baumannii* isolates and 25% clonality of incident ESBL-producing isolates [17], our study did not show significant strain-relatedness or isolate clonality in non-hospitalized, uninjured, healthy Soldiers, regardless of deployed status. The difference in these findings may be attributed to the increased risk of specific MDRO exposure and antimicrobial pressure in healthcare facilities during casualty evacuation in the previous study. No other bacterial species showed broad antimicrobial resistance in our study. While increased colonization rates could explain the increase in ESBL-producing isolates recovered from combat casualties, a comparison of 465 *E. coli* isolates originating from combat-injured patients in Iraq, Afghanistan and the US to isolates in this study found no matching PFGE patterns (data not shown).

The finding of asymptomatic, healthy people colonized with MDR *E. coli*, despite minimal contact with the healthcare system, is concerning. Although numerous studies have reported community associated ESBL-producing *E. coli* urinary tract infections, most have some risk factor for a MDR-GNB infection: genitourinary pathology, previous bacterial infections, prior intravenous antibiotic treatments, and hospitalization in the previous 12 months [5,18]. A study from Madagascar reported a 10% stool colonization rate among healthy people, but the study reported numerous MDR bacterial species (e.g. *E. coli, Klebsiella pneumoniae, Enterobacter cloacae,* and *Citrobacter freundii*) and an association of colonization with socioeconomic status [19]. Other studies have reported MDR stool colonization rates between 1-7%; however, these studies included patients with substantial exposure to the healthcare system and not young, healthy patients as observed in our study [20,21].

We noted that non-MDR *E. coli* recovered from Afghanistan-based study participants was associated with higher antimicrobial resistance, with potential clinical implications associated specifically with ampicillin-sulbactam and tetracycline resistance. As has been previously reported in patients with traumatic abdominal wounds, Enterobacteriaceae may demonstrate significant resistance to ampicillin-sulbactam, risking inadequate therapy in up to 50% of patients [22]. This is consistent with our study finding of 54% ampicillin-sulbactam resistance in non-MDR E.coli isolates from the Afghanistan-based participants. This increased antimicrobial resistance might be associated with exposure to doxycycline for malaria chemoprophylaxis. A previous study assessing the impact of tetracycline and doxycycline exposure on stool bacteria showed no increase in pathogen quantities, to include *Klebsiella*, *Enterobacter*, *Pseudomonas*, *Proteus*, *Serratia,* or *E. coli*[23]. Although the bacteria had increased antimicrobial resistance after tetracycline exposure, this was not observed after doxycycline exposure [23]. Other studies have reported increased resistance after doxycycline exposure and some change in gut flora, but not significantly different than prior to therapy [24,25]. We did not identify genes consistently responsible for tetracycline resistance across the isolate spectrum, but those primarily involved were *tet*(A) and *tet*(B), which is similar to what is described in the literature [26-28].

There is also concern that antimicrobial use in animal husbandry practices might be leading to greater resistance. This has been shown in tetracycline-focused studies that linked *tet*(M)*, tet*(A), and *tet*(B) genes to introns and dairy feed [28]. In the current study, it is worth noting that there was greater tetracycline resistance of the non-MDR *E. coli* isolates associated with the *tet*(A) gene, but no clonality was detected by PFGE. There are reports of TEM-1, CXT-M-15 and *tet*(A) genes carried in the same plasmid, raising concern that doxycycline use might not only be selecting its own resistance, but also ESBL-mediated resistance [29,30]. Increased antibiotic use in food production processes may play a role in this microbial epidemiological finding and warrant further investigation.

It is unclear what role the environment played on the MDR colonization rate of personnel deployed to Afghanistan. Although the participants resided in a remote area of Afghanistan, they had more than adequate potable water for all activities, including bathing, cooking and drinking. The study participants likely had minimal to no consumption of local food, as off-base exposure did not correlate with colonization status. It is also unclear if the use of different swab type for MDR pathogen collection, or the delay in laboratory processing of swabs from Afghanistan, impacted the recovery of pathogens. The overall similarity in total number of pathogens recovered between the two study sites, the similar body areas colonized between the two study sites, and data supporting stability of bacteria on swabs for extended periods of time, suggest that the observed differences are valid. It may be of interest to further assess seasonal variation of MDR pathogen colonization, as our study evaluated individuals primarily in spring and summer months, and may not be representative of colonization prevalence during other seasons. Previous studies have shown seasonal variation in GNB infection rates, with increased incidence of *K. pneumoniae, A. baumannii, and E. coli* infections in summer months [31,32]. Further study limitations include the relatively low percentage of female participants (31% in the US-based study population and no females in the Afghanistan-based population), along with the lack of other potentially relevant demographic data, such as race. These limitations may preclude generalization of our findings to other populations.

Finally, the current study identified a point prevalence of MDR colonization in geographically distinct military populations. Prospective studies on military service members prior to deployments, and prior to malaria chemoprophylaxis/antibiotic exposure, are needed to determine the true incidence of colonization with potential pathogens over time. This study identified antimicrobial resistance patterns that may have implications on clinical treatment decisions, particularly in light of recent literature reports on β-lactamase inhibitor use for ESBL-producing bacteria. For example, we identified MDR *E. coli* colonization isolates that show relative susceptibility to piperacillin-tazobactam, but resistance to ampicillin-sulbactam. These findings would seem consistent with a recent study suggesting clinical efficacy against ESBL-producing *E. coli* bloodstream infections with these antimicrobial agents [33]. The findings in the current study may also impact the choice of early empiric antimicrobial therapy for traumatic injuries during war and possibly humanitarian/disaster relief missions, which may have similar post-injury infection risks. Historically, early antimicrobial therapy in these environments has included agents without enhanced coverage against ESBL-producing bacteria, due to concern for further selection of antimicrobial resistance, and the fact that we have not typically encountered those resistant pathogens early after injury [34]. The recommendation to avoid broader early antimicrobial therapy might need to be reconsidered, especially for injuries involving the perineal and perirectal areas, as these are associated with higher ESBL-producing *E. coli* colonization rates than other body regions.

## Conclusions

We found a 5.5-fold increased rate of community-onset MDR *E. coli* colonization among US military personnel deployed to Afghanistan despite subjects being completely healthy, having only routine acute healthcare exposure (not related to injury or infection) and only minimal antimicrobial exposure (for malaria prophylaxis). Community-associated colonization with other resistant GNB was not observed. Increased colonization rates might explain the increased recovery of ESBL-producing *E. coli* from infections in combat casualties from this geographical region. Further studies should seek to determine the cause and increasing rates of MDR *E. coli* colonization during deployment to Afghanistan. Continued diligence is needed to monitor the changing epidemiology of MDR-GNB and identify causal associations. Finally, continued investigation of the relevance of colonization without active infection is needed to ensure decolonization strategies provide overall benefit without selecting for other resistant pathogens.

## Abbreviations

MDR: Multidrug-resistant; GNB: Gram-negative bacteria; ESBL: Extended-spectrum beta-lactamase; PFGE: Pulsed-field gel electrophoresis; PFT: Pulsed-field type; CLSI: Clinical and Laboratory Standards Insitute; FDA: Food and Drug Administration; MIC: Minimum Inhibitory Concentration; ED: Emergency department.

## Competing interests

The authors declare that they have no competing interests.

## Authors’ contributions

Study design, Data Collection, Data Analysis, Manuscript Development, Writing Manuscript: TJV, DWC, KM, MLL,CKM. Data Collection, Manuscript Development: JPC, EAR, CCT, WCZ, CHC, XU, KAC, KSA, MLB. All authors read and approved the final manuscript.

## Disclaimer

The views expressed herein are those of the authors and do not reflect the official policy or position of the Department of the Army, Department of the Air Force, Department of Defense, or the US Government. The authors are employees of the US government. This work was prepared as part of their official duties and, as such, there is no copyright to be transferred.

This work was supported by the Armed Forces Health Surveillance Center including the Global Emerging Infectious System.

## Pre-publication history

The pre-publication history for this paper can be accessed here:

http://www.biomedcentral.com/1471-2334/13/68/prepub
